# Passage and concentration-dependent effects of Indomethacin on tendon derived cells

**DOI:** 10.1186/1749-799X-4-9

**Published:** 2009-04-02

**Authors:** Emad Mallick, Nanette Scutt, Andy Scutt, Christer Rolf

**Affiliations:** 1 Sheffield Centre Of Sports Medicine, School Of Medicine & Biomedical Sciences, Beech Hill Road, Sheffield, S10 2RS, UK

## Abstract

**Background:**

Non-steroidal anti-inflammatory drugs (NSAID) are commonly used in the treatment of tendinopathies such as tendonitis and tendinosis. Despite this, little is known of their direct actions on tendon-derived cells. As NSAIDs have been shown to delay healing in a number of mesenchymal tissues we have investigated the direct effects of indomethacin on the proliferation of tendon-derived cells.

**Results and Discussion:**

The results obtained were dependent on both the type of cells used and the method of measurement. When measured using the Alamar blue assay, a common method for the measurement of cell proliferation and viability, no effect of indomethacin was seen regardless of cell source. It is likely that this lack of effect was due to a paucity of mitochondrial enzymes in tendon cells.

However, when cell number was assessed using the methylene blue assay, which is a simple nuclear staining technique, an Indomethacin-induced inhibition of proliferation was seen in primary cells but not in secondary subcultures.

**Conclusion:**

These results suggest that firstly, care must be taken when deciding on methodology used to investigate tendon-derived cells as these cells have a quite different metabolism to other mesenchymal derive cells. Secondly, Indomethacin can inhibit the proliferation of primary tendon derived cells and that secondary subculture selects for a population of cells that is unresponsive to this drug.

## Introduction

Non-steroidal inflammatory drugs (NSAIDs) are commonly used for the treatment of a number of musculoskeletal sports injuries including the inflammation of tendons and ligaments. A number of studies have however, suggested that NSAIDs may delay soft tissue healing although the exact mechanism of action for this is unknown [1-4]. Some *in vitro *investigations on the effects of NSAIDs on tenocytes have been performed [5-9]. However, they have used limited dose-ranges of NSAIDs and subcultures of tenocytes. We have previously argued that sub-culturing tenocytes selects for rapidly proliferating population of cells and is not necessarily representative of the situation found *in vivo *where the majority of cells are non-proliferative [10,11]. In contrast, primary cultures of tenocytes contain all of the cells originally present in the tendon, both differentiated and undifferentiated, and would therefore seem likely to be a more realistic model of tendon metabolism. We have therefore investigated the effects of NSAIDs on both primary tenocytes and secondary and tertiary subcultures of the cells.

## Methods

Isolation and culture of tendon derived cells: Tendon derived cells (TDC) were obtained from the tail tendons of 200 g male Wistar rats. Rat-tail tendons were chosen because they can be obtained in sufficient quantities to allow the extensive use of primary cells. Although they are not completely relevant to human pathologies they show similar age-related changes in their biomechanical properties to other tendons and in this laboratory rat tail TDC behave similarly to cells derived from other tendons; human and rat. The rats were maintained according to UK home office regulations and killed by a schedule 1 method. The tendons were dissected free from the tails and the TDC freed from the tendons by digesting for 18 h at 37°C in 1 mg/ml crude collagenase in culture medium.

After digestion the cells were washed, resuspended and viable cells determined.

The cells were then used immediately in primary high-density cultures or plated out for secondary cultures.

Primary or secondary cells were plated out in 24 well plates at a density of 10,000 cells per well in DMEM containing 10% FCS, penicillin/streptomycin and glutamine. The cells were treated with indomethacin (0.1 nm – 100 uM) for 6 days. The cultures were then stopped and cell number determined by either Alamar blue assay, methylene blue assay or by direct counting using a Guava PCS.

### Alamar blue assay

At the end of the culture period 50 μL of Alamar blue was added to the cultures, which were then incubated at 37°C for further four hours. Cell number was then determined by analysing the supernatants spectrophotometrically at 570 and 600 nm.

### Methylene blue assay

The cells were fixed with cold ethanol and then washed with borate buffer (pH 8.8, 20 mM). Cells were then stained with methylene blue (1 mg/ml in borate buffer) for 30 minutes after which they were washed three times with borate buffer. The dye was then eluted with 1% HCl in ethanol and cell number determined by measuring the absorbance at 650 nm.

### Guava PCS

The cells were diluted 1 in 10 in Guava Viacount reagent (containing 7-amino-actinomycin D) and cell number and viability determined using a guava personal cytometry system according to the manufacturer's instructions.

Data handling and statistical analyses: Data are presented as group mean ± standard deviation. At least 3 replicates of each experiment were performed, and the results presented in the figures are representative of these. For each variable, effects across treatment groups were compared with one-way analysis of variance (ANOVA). If the overall difference was significant, multiple comparisons were performed between groups using Tukey's test. Differences are considered significant at a probability <0.05 on a two-tailed test.

## Results

**Figure 1:**
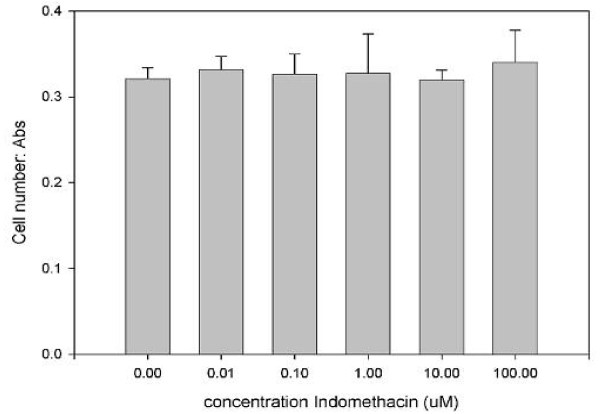
**This graph shows the relationship between increasing Indomethacin concentration and cell number of primary tendon derived cells as measured by Alamar Blue**.

**Figure 2:**
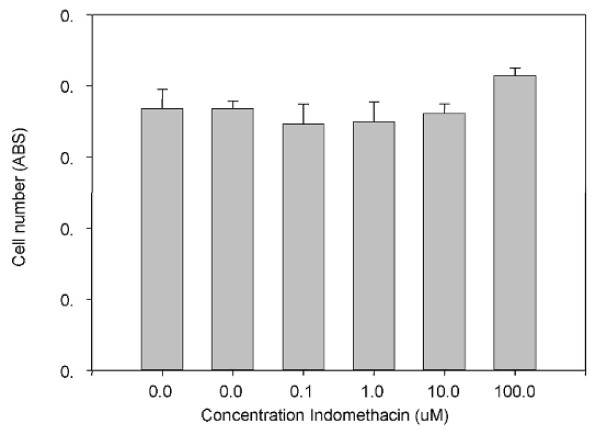
**This graph shows the relationship between increasing Indomethacin concentration and cell number of secondary tendon derived cells as measured by Alamar Blue**.

Initial experiments studying the effects of indomethacin on tendon derived cell proliferation were carried out using the commonly used Alamar blue assay. However, although on visual examination an effect was evident, no effect was seen after analysis of the cell supernatants (Figure 1 & Figure 2).

**Figure 3:**
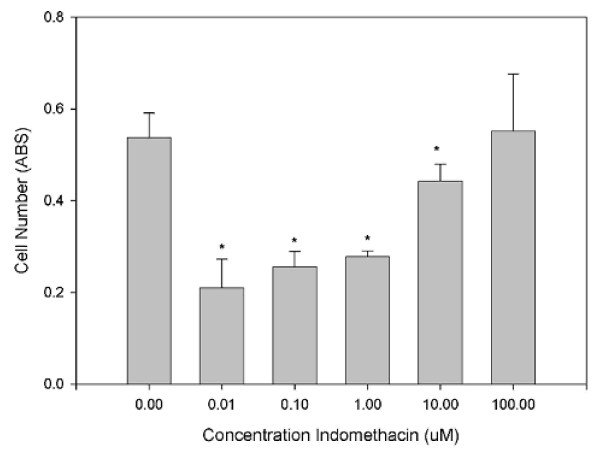
**This graph shows the relationship between increasing Indomethacin concentration and cell number of primary tendon derived cells as measured by Methylene Blue**.

**Figure 4:**
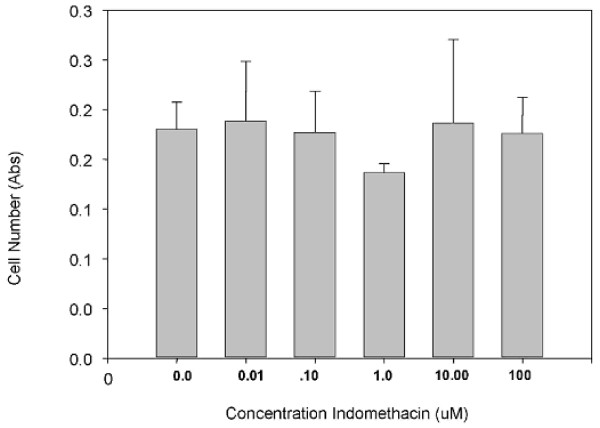
**This graph shows the relationship between increasing Indomethacin concentration and cell number of Secondary tendon derived cells as measured by Methylene Blue**.

We therefore adopted the methylene blue assay to determine tendon-derived cell numbers. Although this assay has the disadvantage of detecting both live and dead cells it is thoroughly reliable and accurate. Using this assay, it was found that treating primary cells with indomethacin lead to a dose related inhibition of cell proliferation. However, rather unexpectedly the relationship was negative with the greatest inhibition being seen at 10 nM and essentially no effect at 100 μM (Figure 3). In secondary subcultures of tendon-derived cells, the cells became relatively refractive to treatment with indomethacin showing no significant effect at any concentration (Figure 4) and then by the third subculture a biphasic stimulation of proliferation were seen with a maximum at 1–10 μM (data not shown).

**Figure 5:**
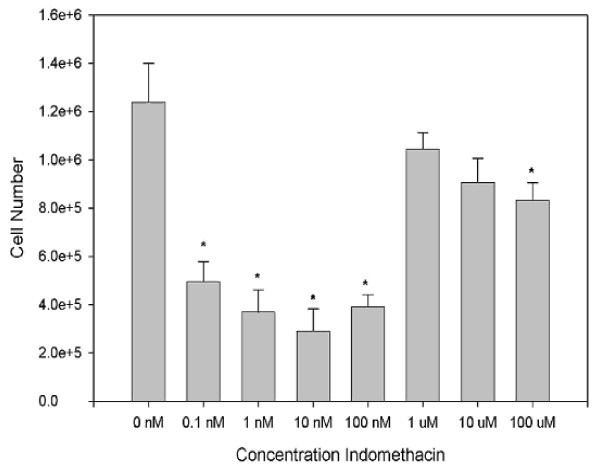
**This graph shows the relationship between increasing Indomethacin concentration and cell number of primary tendon derived cells as measured by direct cell counting methods**.

Because of the somewhat unexpected inhibition of proliferation with low concentrations of Indomethacin, this work was repeated using direct counts of cell number and viability (as measured by 7-amino-actinomycin D uptake). Although the concentration relationship was not as linear as with the methylene blue assay, this too showed a significant decrease in cell number at lower concentrations of indomethacin whereas treatment with high concentrations had essentially no effect (Figure 5). Also the results with secondary sub culture of tenocytes were similar to methylene blue with no effect of indomethacin on proliferation. We did not repeat this with tertiary subculture of tenocytes.

**Figure 6:**
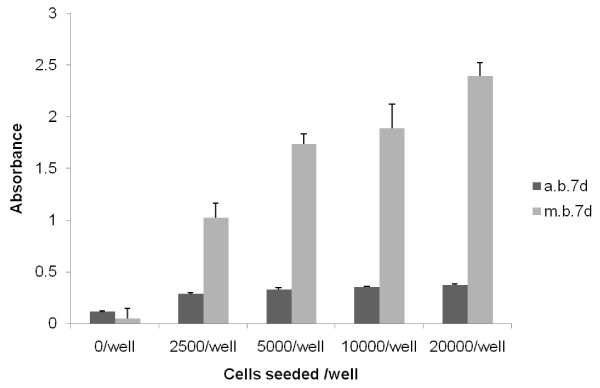
**Comparison of absorbances of Alamar blue and methylene blue obtained after culture of rat tail tendon derived cells at varying concentrations for 2 and 7 days**. Data points are mean of 3 wells. Results were significant between cell numbers seeded for both dyes at both time points using ANOVA.

**Figure 7:**
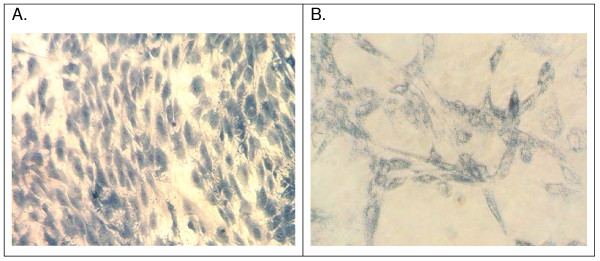
**Staining of tendon derived cells with A, methylene blue or B, MTT**. Monolayer cultures of rat tail tendon cells were grown to confluence, then stained with these substances as described in materials and methods. Following staining cultures were photographed. Staining with methylene blue produces even staining whereas staining with MTT produces patchy results with some cells staining strongly and others barely staining at all.

The lack of response when measuring the tenocytes cell number using Alamar Blue was somewhat unexpected as this is a well established method of determining cell number and is used with a wide variety of cell types. An experiment relating cell number and absorption revealed this to be a cell dependent problem as Methylene blue staining gave rise to a strong cell-dependent increase in staining whereas the response using Alamar blue was essentially flat with very little response (Figure 6). In comparison, mesenchymal stem cells produce very similar curves regardless of whether they are stained with Methylene blue or Alamar blue (data not shown). To try and determine the cause of this we stained the cells with 3-(4,5-Dimethylthiazol-2-yl)-2,5-diphenyltetrazolium bromide (commonly known as MTT) which is metabolized from yellow MTT to an insoluble purple formazan by a similar mechanism to Alamar blue. It was found that whereas Methylene blue stained all of the cells evenly throughout the cultures, it was evident that there were 2 populations of cells in the tenocyte cultures, one staining strongly with MTT and the other barely staining at all (Figure 7).

## Discussion

Tendon injuries produce considerable morbidity, and the disability that they cause may last for several months despite what is considered appropriate management [12]. The basic cell biology of tendons is still not fully understood, and the management of tendon injury poses a considerable challenge for clinicians [12]. Even though histological studies have shown absence of acute inflammatory cells in chronic tendonitis, NSAIDs are used commonly for managing tendon injuries [13-16]. Clinical studies indicate that they decrease the pain, tenderness and stiffness associated with acute soft tissue injury 3(4). The effect of NSAIDs on tenocytes however, remains debatable. Studies with NSAIDs have shown both beneficial [7,17] and harmful effects on tenocytes [6,8]. As the tenocyte is the major cell type in tendons and is responsible for the production and maintenance of extra cellular matrix, we investigated the effect of Indomethacin, commonly used NSAIDs for tendon injuries on tenocyte proliferation. Of particular interest to our study was the effect of primary and sub culture of tenocytes and different cell counting methods for tenocytes.

Tenocyte culture provides a uniform, controlled environment in which to study the in vitro effects of Indomethacin, with limitation of other uncontrolled variables and avoidance of the confounding influences that are present in animal models [18]. The results of our in vitro experiments showed that Indomethacin had a dose related effect on tenocyte proliferation. Indomethacin reduced the number of human tenocyte cells at a dose between 0.1 nM to 100 nM. However at higher doses it did not inhibit tenocyte proliferation. This is in contrast to some studies showing inhibition of tenocytes with therapeutic doses of Indomethacin and hence producing a negative effect on healing tendons [6,8]. Other studies have shown beneficial effect on tenocyte healing by increasing the tensile strength to failure [5,7,17]. This is due to increased maturation of collagen fibrils during healing. However it is still debatable whether this beneficial effect is due to proliferation of tenocytes or increased production of collagen and maturation. Our study indicating that Indomethacin in therapeutic doses did not inhibit tenocyte proliferation has clinical relevance. This would be beneficial in tendon healing and repair as tenocyte is the major cell type in tendons and is responsible for the production and maintenance of extracellular matrix.

The negative dose response effect of Indomethacin on tendon derived cell proliferation remains a conundrum and we have no explanation to this. It should however be noted that serum levels of Indomethacin generally reach a peak of 1–10 μg/ml after administration and due to the poor blood supply in tendons, it is likely that tenocytes are exposed to concentrations even lower than this. These concentrations are well within the range at which the above effects were seen in vitro but did not go any way towards explaining the lack of effect at higher concentrations.

Dissimilar culture conditions as in vivo or in vitro, differing species and even changes in cell culture conditions of the same species have shown different tenocyte behaviour [5-7,17-20]. It can thus be stated that interaction between tenocytes and NSAIDS is influenced by different factors, leading to opposing results. It has been previously argued that sub culture of mesenchymal cells do not reciprocate in vivo results [11]. However this effect has not been studied or documented before, with tenocytes. We considered this as an important area of investigation and looked at role of culture conditions on effect of Indomethacin on tenocytes. We noted that sub culture of cells did not show similar results as primary tenocytes. The dose dependent effect of Indomethacin was only seen on primary tenocytes and sub culture of cells did not show this effect. Previous studies have shown Indomethacin to cause both inhibition and proliferation of tenocytes in in vitro studies [6,10,14,17]. However these studies do not clearly indicate if only primary or subcultures of cells were used. Also it has not been mentioned previously if such effect was seen. We postulate that this effect seen in our study may be due to the selection of a highly proliferative population of tenocytes that is present in the primary digest only at low levels which subsequently overgrow the slower growing cells in later passages. We consider this an important finding, as studies, which have used sub culture of cells, need to be reviewed with some caution.

Various cell-counting methods are used to measure tenocyte proliferation. Initial experiments studying the effects of Indomethacin on tendon derived cell proliferation were carried out using the commonly used Alamar blue assay. However, although on visual examination an effect was evident, no effect was seen after analysis of the cell supernatants. This conundrum was subsequently explained by the finding that tendons appear to contain 2 subpopulations of cells; one subpopulation with apparently normal metabolic activity and a second subpopulation of cells with low levels of mitochondrial enzymes and subsequently a low oxidative metabolism. As the Alamar blue assay is dependent on mitochondrial enzyme activity for the reduction of the dye resazurin to resorufin, in cells without these enzymes, no effect was seen. This was confirmed by the use of MTT which is metabolized by the same enzymes to produce a purple product and it was clearly demonstrated that 2 subpopulations of cells exist; one staining intensely indicating a high metabolic activity and one stained relatively mildly indicating a low metabolic activity. This is entirely consistent current knowledge regarding the metabolic activity of tenocytes and tenoblasts. Tenoblasts are known to contain relatively high numbers of mitochondria and tenocytes very few suggesting comparatively high and low oxidative metabolism respectively [21]. This would in turn that the response seen in primary cells is due to the presence of mature tenocytes in the cultures which would have been lost on subsequent passage. As tenocytes are thought to be non-proliferative it is obviously unlikely that this is due to an inhibition of proliferation. One possibility is a toxic effect on specific to tenocytes but not tenoblasts however, we saw no evidence of excessive cell death in these cultures. Another possibility is that the tenocytes play some form of regulatory role controlling the proliferation of the proliferative tenoblasts as is thought to be the case for osteocytes and osteoblasts [22]. This must however remain speculative until further evidence is available.

One consequence of these data is that investigations using Alamar blue or MTT to assess tenocytes numbers should be interpreted with some caution. We therefore adopted the methylene blue assay to determine tendon-derived cell numbers. Although this assay has the disadvantage of detecting both live and dead cells it is thoroughly reliable and accurate. Using this assay, it was found that treating primary cells with indomethacin led to a dose related inhibition of cell proliferation. In secondary subcultures of tendon-derived cells, the cells became relatively refractive to treatment with indomethacin showing no significant effect at any concentration (Figure 4). Because of the somewhat unexpected inhibition of proliferation with low concentrations of Indomethacin, this work was repeated using direct counts of cell number and viability (as measured by 7-amino-actinomycin D uptake). Although the concentration relationship was not as good as with the methylene blue assay, this too showed a significant decrease in cell number at lower concentrations of indomethacin whereas treatment with high concentrations had essentially no effect (Figure 5).

The cause of this rather unexpected dose/effect relationship is at present unclear. Indomethacin is thought to act principally by the modulation of arachidonic acid metabolism via the cyclooxygenase and lipoxygenase pathways thus blocking the production of prostaglandins and HETEs/leukotrienes respectively. The relationship is however not simple as indomethacin has been shown to stimulate [23] and inhibit [24] the production of lipoxygenase metabolites. This differential effect is likely to be both tissue and concentration dependent and is further complicated by the possibility that blocking one pathway is likely to shunt metabolites down the other. No drugs are 100% specific for any one particular mechanism of action and indomethacin is no exception giving rise to a number of effects unrelated to cyclooxygenase [25]. Lastly, endocannabinoids are now known to have a number of peripheral effects on connective tissue [26] and preliminary investigations in this laboratory indicate that this is also the case in tendons and ligaments. In relation to this, indomethacin has been shown to inhibit the enzyme fatty acid amide hydrolase (FAAH), which metabolizes endocannabinoids [27]; inhibition of FAAH would therefore result in the build of levels of endocannabinoids. The situation is therefore highly complex and is unlikely to be unraveled until further insights into the relative roles of prostaglandins, leukotrienes and endocannabinoids in tendons are obtained.

It should be stressed that in this study we have only investigated the effects of indomethacin on cell proliferation and therefore no firm conclusions can be made regarding the effects of indomethacin on other metabolic processes. There have been few studies on tendon cells monitoring both proliferation and collagen accumulation, however, we have found that collagen accumulation in tendon cells normally parallels cell number and that specific levels of collagen synthesis remain largely unchanged [28-31]. This would suggest that indomethacin would probably produce similar effects on matrix protein synthesis to those described above, although this obviously requires confirmation.

## Conclusion

In conclusion, these data show that primary tenocytes respond to Indomethacin differently compared to secondary and tertiary subcultures. This may be due to the selection of a highly proliferative population of tenocytes that is present in the primary digest only at low levels which subsequently overgrows the slower growing cells in later passages. This would suggest that where possible *in vitro *investigations into tenocyte metabolism should preferentially be performed using primary cells and that results obtained using subcultures should be viewed with some caution.

Furthermore we have shown that because of their lower metabolic rate tendon derived cells cannot sufficiently metabolise Resazurin, the dye used in the Alamar blue assay. Because of this we would suggest that this essay is not appropriate studying proliferation in tendon derived cells and that an alternative, such as the methylene blue assay, should be used.

## Competing interests

No author in any form has received any financial support or reward for this study. Also there are no non – financial competing interests.

## Authors' contributions

All authors have significantly contributed in the conception and design, or acquisition of data, or analysis and interpretation of data and have been involved in drafting the manuscript or revising it critically for important intellectual content; and have given final approval of the version to be published.
